# Development of an acute medical unit to optimize patient flow and early discharges in a tertiary care hospital in the United Arab Emirates

**DOI:** 10.1186/s12913-022-08746-0

**Published:** 2022-11-29

**Authors:** Thana Harhara, Halah Ibrahim, Waqar H. Gaba, Ashraf M. Kamour

**Affiliations:** 1 Department of Medicine, Sheikh Khalifa Medical City, PO Box 51900, Abu Dhabi, United Arab Emirates; 2 Khalifa University College of Medicine and Health Sciences, Abu Dhabi, United Arab Emirates

**Keywords:** Acute medical unit, Length of stay, Emergency department crowding, Multidisciplinary team

## Abstract

**Background:**

Hospitals worldwide are seeing an increased number of acute admissions, with resultant emergency department (ED) crowding and increased length of stay (LOS). Acute Medical Units (AMUs) have developed throughout the United Kingdom and other Western countries to reduce the burden on EDs and improve patient flow. Limited information is available on AMUs in the Middle East. The purpose of this study is to describe the development of the first AMU in the United Arab Emirates (UAE) for general medical patients and its impact on LOS, early discharges, ED boarders, and readmission rates.

**Methods:**

We established a consultant-led AMU in a tertiary hospital in the UAE. A retrospective comparative review of all general medical admissions to the AMU between August 1, 2020 and December 31, 2020 and all admissions to the traditional medical wards between August 1, 2019 and December 31, 2019 was conducted.

**Results:**

The average LOS reduced from 10 to 5 days (95% CI [4.14–6.25], p < 0.001) after the introduction of AMU. Early discharges increased by 22%. The number of outliers and number of patients boarding in ED reduced significantly (111 in 2019 vs. 60 in 2020, p < 0.05; 938 in 2019 vs. 104 in 2020, p < 0.001 respectively), with a decrease in ED waiting time from 394 min to 134 min (95% CI [229.25–290.75], p < 0.001). There was no increase in 30-day readmission rates.

**Conclusion:**

Restructuring the system of care can reduce LOS, overcome discharge barriers and improve patient flow. Similar units can be developed in hospitals throughout the UAE and the region to reduce LOS and improve patient flow through acute care units.

## Background

Hospitals worldwide are providing care for an increasing number of acutely ill patients due to aging populations with multiple comorbidities and complex care needs [1]. Limited hospital beds, combined with medical and social barriers to timely discharges, have led to overcrowding of emergency departments (ED) and increased waiting times for inpatient beds [1, 2]. ED crowding is associated with inefficient care delivery and higher rates of medical errors and complications [3]. The lack of bed availability also results in patient admissions to clinically inappropriate wards, often referred to as outliers, raising safety concerns and potentially compromising care [4, 5]. Several strategies have been suggested to improve system efficiency and optimize the care and flow of patients [6]. The development of acute medical units (AMU) for rapid multidisciplinary assessment of acutely ill medical patients has shown favorable results in several countries [7, 8].

The Royal College of Physicians defines the AMU as “a dedicated facility within a hospital that acts as the focus for acute medical care for patients who present as medical emergencies ” [9]. AMUs provide expedited multidisciplinary assessment and care for the first 24–72 hours of a patient’s hospital admission [9, 10]. A systematic review across five countries revealed that adopting the AMU model helped reduce length-of-stay (LOS) without adversely impacting readmission rates [11]. AMU implementation can lead to more efficient bed utilization, resulting in decreased ED waiting times and fewer medical patients admitted to non-medical beds [7, 12]. AMUs have also been shown to improve the quality of care by expediting diagnostic and therapeutic interventions and fostering multidisciplinary teamwork and inter-professional collaboration [13].

The AMU model has gained popularity worldwide, with hospitals in Australia, New Zealand, the United Kingdom, and other European nations implementing AMUs instead of, or in addition to, traditional medical wards [10, 13]. Most of the literature, however, derives from Western countries, with little known about the prevalence or outcomes of AMUs in other parts of the world, particularly in developing healthcare systems. In 2015, Hamad General Hospital in Qatar established a medical assessment unit, with early results showing improvement in patient flow [14]. To our knowledge, there are no other published studies on the implementation of AMUs in the Middle East. As countries in the region work to improve the quality and efficiency of healthcare delivery, studies evaluating the financial and patient care impact of health service interventions are necessary. This manuscript describes the development of the first AMU in the United Arab Emirates (UAE) for general medical patients, highlighting the impact of introducing an AMU on LOS, ED boarding time, medical patient boarding in non-medical units, and 30-day readmission rates.

## Methods

### Setting

The UAE is a small nation in the Middle East, formed in 1971 as a federation of 7 emirates. The total UAE population is 9.9 million [15]. Abu Dhabi is the largest emirate occupying 84% of the national landmass territory. 16]. Over the past two decades, the UAE government has made substantial investments in the healthcare system, with a focus on the quality and accessibility of care provided. This has coincided with an aging society and increased prevalence of cancer and other non-communicable diseases [17]. At present, government hospitals are the largest providers of health professional education and healthcare delivery in the country. Sheikh Khalifa Medical City (SKMC) is a 586-bed government tertiary care center in Abu Dhabi, the capital of the UAE, and the second most populous city in the country, with a population of 2.9 million [16]. The hospital serves a diverse patient population and has emergency, ambulatory, and inpatient services.

Historically, the internal medicine department at SKMC was comprised of six medical wards, with a total of 160 patient beds. All non-critically ill medical patients were admitted to the internal medicine service through the ED, from the outpatient clinics, or through direct transfers from local hospitals. The hospital also has closed intensive care, coronary care, and stroke units, each managed by the relevant specialty. Delays in diagnostic interventions and subspecialty consultations and inefficient hospital discharges often led to prolonged LOS on the internal medicine service, resulting in an average of 7% of our patients being admitted to non-medical wards due to capacity strains and lack of beds in the designated internal medicine units. In 2019, the average LOS in the inpatient medical ward was 15 days.

**Fig. 1:**
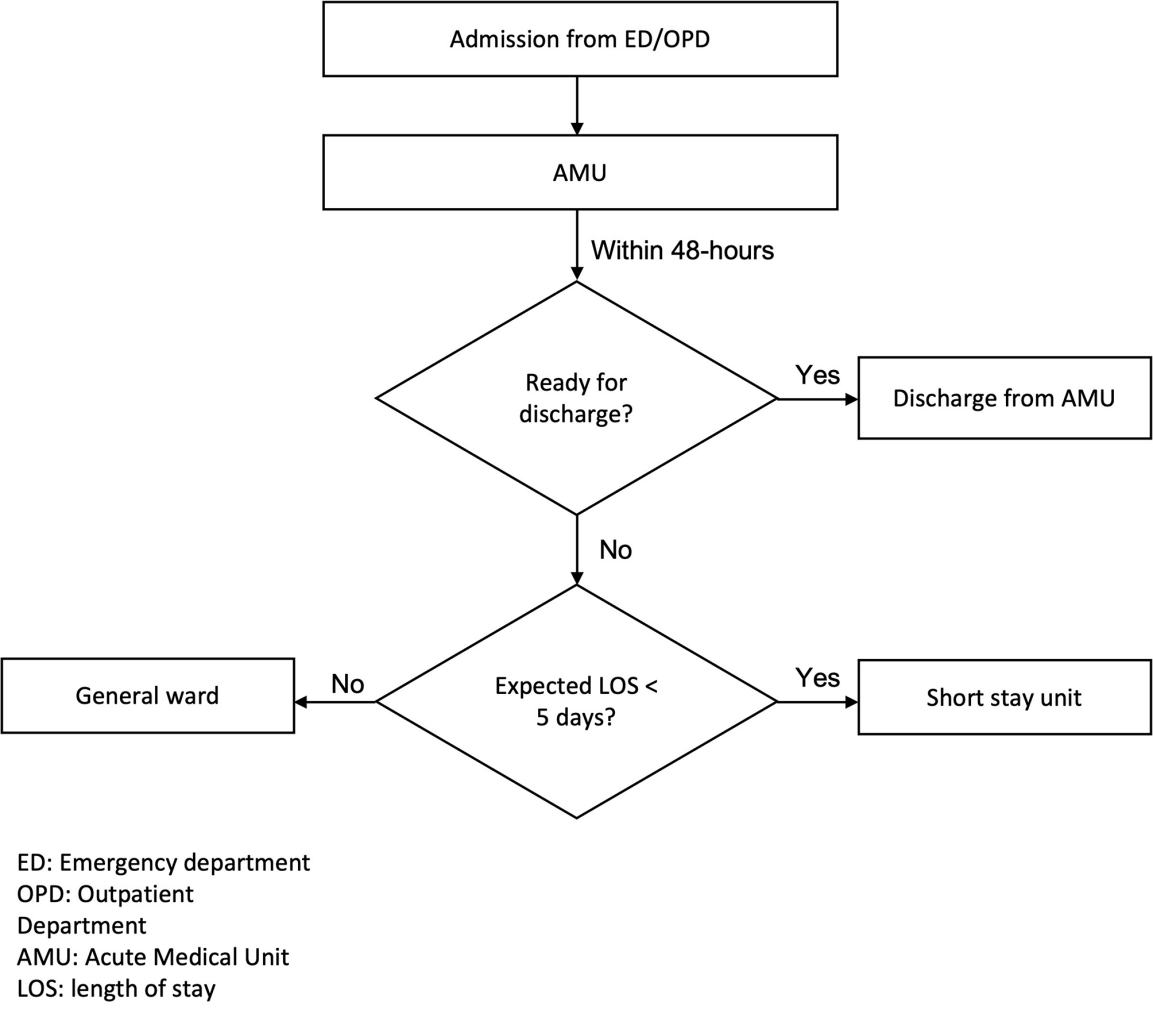
Patient Flow through AMU

In August 2020, an AMU was established in the internal medicine department. The goal of the unit was to optimize efficiency and improve the quality of care. All medical patients who required inpatient care were admitted to the AMU for the first 24–48 h of hospitalization (Fig. 1). The unit consisted of 21 beds, each equipped with telemetry monitoring, and six were assigned as high-dependency beds. The unit was operational 24-hours, 7-days a week. Existing staff were redeployed from the medical wards and schedules were adjusted accordingly. Two medical teams were based in the AMU, each consisting of a senior internal medicine consultant physician, a senior resident, and 2 to 3 junior residents. The physicians worked 12-hour shifts daily (8 am to 8 pm), with after-hour coverage provided by the inpatient on-call team. The extended-hour coverage offered early access to senior consultant input.

Surgical and medical subspecialties and allied health professionals were assigned to the AMU and made early morning ward rounds to proactively provide input and expedite diagnostic and therapeutic interventions. The AMU also had a dedicated case manager to aid in the early identification of discharge needs and disposition planning. Multidisciplinary huddles with physicians, nurses, physiotherapists, dieticians, social workers, pharmacists, and case managers took place daily at 8 am. Follow-up huddles with the charge nurse, case manager and the medical team took place after rounds at 11 am and 3 pm.

After stabilization in the AMU, patients with an expected LOS exceeding 48 h were transferred to a short stay unit, and those who required longer hospitalizations (more than 5 days) were transferred to a traditional medical ward. Discharges and transfers were identified early in the day to ensure bed availability in the AMU for new admissions.

### Data Collection and Analysis

A retrospective chart review of all admissions to the AMU was conducted between August 1, 2020 and December 31, 2020, both days inclusive. All general medicine admissions between August 1, 2019 and December 31, 2019 were also reviewed as a base year before AMU establishment, during which patients were admitted to the traditional medical wards. Data were obtained from the electronic medical record to review LOS, rates of early discharges within 48-hours of admission, and allocated wards upon hospital admission. The authors also analyzed the total number of medical patients boarding in the ED and their ED LOS, and determined 30-day readmission rates. Case mix index (CMI) based on the Medicare Severity diagnosis-related group coding system was used to reflect the illness severity of the patients [18]. Descriptive and comparative statistical analysis was performed using the statistical software package MedCalc→ 20.115 [19]. Descriptive statistics were used to calculate changes in variables pre-and post-intervention. Chi-square tests and Fisher’s exact test were used to compare categorical variables. Odds ratios (OR) were calculated where appropriate. Statistical significance at p < 0.05 was assumed throughout. Of note, all patients with COVID-19 infection were admitted to a separate infectious disease ward and were excluded from our analysis. The study was approved by the Sheikh Khalifa Medical City Research Ethics Committee (RS-732).

## Results

Table 1 summarizes the results. A total of 1755 patients were admitted to internal medicine during the study period, of which 23.8% were patients with COVID-19 infection, who were admitted directly to isolation wards and excluded from the study. The top five admission diagnoses to AMU are listed in Table 2. Of the 1338 patients admitted to AMU, 41% were discharged within 48-hours, compared to 19% early discharges in 2019 (p < 0.001). Of the total AMU admissions, 590 (44%) were discharged directly from the AMU, 349 (26%) were transferred to and subsequently discharged from the short stay unit, and all other patients were transferred to the regular medical wards. The average LOS was 10 days for the period of August to December 2019 compared to 5 days for the period of August to December 2020 (95% CI [4.14–6.25], p < 0.001). There was a statistically significant reduction in the total number of medical patients boarding in the ED (938 in 2019 vs. 104 in 2020, p < 0.001) after the implementation of AMU. The average LOS of medical patients boarding in the ED reduced from 394 min to 134 min (95% CI [229.25–290.75], p < 0.001) after AMU establishment. There was also a statistically significant reduction in the number of patients admitted outside the designated medical wards (111 in 2019 vs. 60 in 2020, p < 0.05). There was no significant difference in 30-day readmission rates or mortality rates.

**Table 1:** Patient demographics and outcomes before and after AMU implementation

Variable	Pre-intervention	Post-intervention	Odds Ratio	p-value
Mean age (years)	53	51		< 0.01
Gender: Male	848 (52.5%)	795 (59.4%)	0.76	< 0.001
Female	766 (47.5%)	543 (40.6%)		
**Outcome**				
Total number of admissions	1614	1338		
Number of patients discharged within 48 h	307	551	2.98	< 0.001
30-day readmission	51	54	0.78	0.230
All-cause mortality	60	38	1.32	> 0.05
Average case mix index	1.5	1.4		
Average length of stay (days)	10	5		< 0.001
Number of patients boarding in ED	938	104	16.46	< 0.001
Average boarding time in ED (minutes)	394	134		< 0.001
Number of outliers	111	60	1.57	< 0.05

**Table 2:** Top 5 admission diagnoses to the acute medical unit

Diagnosis	%
Sepsis	8.9
Gastrointestinal hemorrhage	4.6
Acute kidney injury	4.6
Pneumonia	4.3
Sickle cell crisis	4.3

## Discussion

In this study of the establishment and early outcomes of the first AMU in the UAE, the authors found that the aggregation of acute medical admissions on a dedicated ward, with early assessment and treatment by a multidisciplinary team, was successful in reducing LOS and increasing early discharges without an increase in readmission rates or deaths. The intervention also successfully reduced the number of patients boarding in the ED and the number of outliers. These results are similar to international studies that show AMUs reduce LOS [7, 8]. While patient-level characteristics influence LOS, research has shown that organizational factors, including delays in assessments, obtaining diagnostic and medical interventions, and shortages in service coverage, especially on weekends and after-hours, also contribute to discharge delays [20].

There are several potential reasons why the implementation of the AMU has led to an increased number of discharges and a reduction in LOS. Clustering all new medical patients in a single location and assigning a dedicated group of healthcare providers to the unit creates an environment designed to promptly meet the patients’ clinical and non-clinical care needs, focusing on early discharges. Concentrating acute medical admissions in a single ward within a large health center also eliminates unproductive time traveling through the hospital, thereby maximizing the medical team’s time and energy. Further, the consultant-led service delivery in the critical first hours of an admission facilitates early review and clinical decision-making, which supports timely decisions on treatment and disposition. Early input by senior physicians has been shown to positively impact patient outcomes for acute medical admissions [21]. Studies suggest that increased consultant presence is associated with improved outcomes of care, with a reduction in mortality and readmissions and an increase in same-day discharges [22, 23]. However, there is limited evidence on the optimal staffing of AMUs [10]. Medical staffing needs to consider the workload over a 24-hour period, with local service reviews to audit the unit’s performance and validate staffing needs [24]. Research also shows that the provision of multidisciplinary care and rapid assessment of patients improve the quality and efficiency of care [25, 26].

Procedural inefficiencies, including waiting for tests, investigations, and input from consult services, contribute to delays in discharges [27, 28] A study examining causes of delayed discharge from the general medical wards of a metropolitan teaching hospital showed that 48.2% of patients experienced delays waiting for a service that extended their LOS [29]. The AMU received expedited access to imaging and diagnostic testing and interventions, which aided in timely decision-making. Additionally, concentrating all new patients in one geographically defined area helped subspecialties refine their workflows to prioritize new patient assessments and provide prompt consultation services.

Paramount to the discharge process was the dedicated multidisciplinary team huddles that proactively identified discharge barriers and needs, as research has revealed that the lack of timely discharge planning negatively impacts LOS [27]. Interventions that address information sharing, such as those done during the team huddles, can best address discharge delays [20]. The multiple daily meetings ensured smooth coordination of the discharge process and clear communication with all healthcare professionals.

Similar to other studies, the introduction of the AMU in the hospital led to a decrease in the number of patients boarding in the ED and on non-medical wards [13]. The United States General Accounting Office highlights that the inability to transfer a patient from the ED due to the lack of inpatient beds is most the common factor associated with ED overcrowding [6]. Inpatient beds that are occupied by patients awaiting discharges cause bottlenecks in patient flow and create access blocks at multiple levels in the hospital system [30, 31]. By reducing LOS and increasing early inpatient discharges, timely access to acute hospital beds for patients requiring emergency admission was facilitated. The early identification of patients who were expected to exceed the AMU’s 48-hour LOS ensured that a contingency plan was in place to help coordinate transfers into other inpatient wards and reduce ED waiting time for patients waiting for an inpatient bed.

Unplanned patient readmission is a significant patient safety concern. The authors are encouraged that readmission rates did not increase after the implementation of the AMU model, despite the increased early discharge rate and reduction in mean LOS. Senior physician oversight on all aspects of care contributed to appropriate and timely discharge decisions. In addition, the daily multidisciplinary team meetings and active engagement of the dedicated case managers placed a focus on safe discharges, which likely positively impacted readmission rates.

The problem of delayed discharges and increasing LOS is a system-level issue and requires a whole-system approach [32, 33]. Best practices to address discharge delays target practice changes and information sharing [20, 34]. The implementation of the AMU involved both an organizational change by restructuring the internal medicine service to improve patient flow and enhance the system’s efficiency, as well as improved departmental communication to facilitate information sharing and frequent opportunities for interprofessional collaboration. The development of this model required a hospital-wide approach and support from senior leadership. The clinical team had extensive discussions with all stakeholders, including hospital administrators, nursing and bed management representatives, clinical departments, and allied health care teams. Workflows were restructured to streamline the process of admitting all non-critically ill adult inpatients to a designated geographic location. The hospital leadership facilitated converting an existing ward in-close proximity to the emergency and radiology departments into the AMU.

As the UAE faces an aging population with increased prevalence of chronic diseases [17], studies of interventions that increase access and improve the quality of patient care, while reducing healthcare costs are essential. The study’s findings must be viewed in light of some limitations. First, the study was conducted in a single hospital in the UAE, thus limiting generalizability. Second, the overall number of admissions in 2020 was less than in 2019, which may have contributed to the reduction in the number of patients boarding in the ED and other wards. This trend is consistent with changes in hospitalization patterns for patients with non-COVID-19 diagnoses during and after the COVID-19 pandemic [35]. Finally, other interventions addressing patients with a prolonged LOS were conducted during the same time as the introduction of the AMU [34]. However, these interventions focused on complex discharge planning in patients with prolonged hospitalization and did not address early discharges from the AMU. Despite these limitations, this study adds to the literature on AMU and the feasibility of implementing this model in a non-Western healthcare system.

As a result of the model’s success, other departments within the hospital, such as Pediatrics, are now considering developing an AMU. In addition, results of the intervention have been shared in local health conferences and through senior leadership meetings with neighboring healthcare facilities. Several government and private hospitals in Abu Dhabi are currently in the process of adopting the AMU model.

## Conclusion

Restructuring the system of care by implementing an AMU can reduce LOS and overcome discharge barriers while reducing ED crowding and improving patient flow. The AMU model is practical and feasible and can be implemented in hospitals throughout the UAE and the region. Further studies on quality measures in the AMU, healthcare team satisfaction, and the effect of the multidisciplinary huddles on team morale and collaboration are needed.

## Data Availability

The datasets generated and/or analyzed during the current study are not publicly available due to the institution’s policy to code and archive data in a central repository of the hospital, but are available from the corresponding author on reasonable request.

## List of abbreviations

ED: Emergency department
AMU: Acute medical unit
LOS: Length of stay
UAE: United Arab Emirates
SKMC: Sheikh Khalifa Medical City
